# Near-maximum microwave absorption in a thin metal film at the pseudo-free-standing limit

**DOI:** 10.1038/s41598-022-23119-7

**Published:** 2022-11-01

**Authors:** Mahsa Haddadi. M, Bamadev Das, Jeeyoon Jeong, Sunghwan Kim, Dai-Sik Kim

**Affiliations:** 1 Department of Physics and Quantum Photonics Institute, Ulsan National Institute of Science and Technology (UNIST), Ulsan, 44919 Republic of Korea; 2 Department of Physics, Institute of Quantum Convergence Technology, Kangwon National University, 1 Gangwondaehak-gil, Chuncheon-si, 24341 Gangwon-do Republic of Korea

**Keywords:** Optics and photonics, Optical physics

## Abstract

Electromagnetic absorbers based on ultra-thin metallic film are desirable for many applications such as plasmonics, metamaterials, and long-wavelength detectors. A metallic film will achieve a maximum 50% of electromagnetic wave absorption, frequency independent, at a thickness defined by its conductivity, typically in the sub-Angstrom range for good metals if bulk conductivity is maintained throughout. This makes it extremely difficult to obtain substantial absorption from thin metal films, in contrast to 2D materials such as graphene. Luckily, however, from a practical point of view, metal conductivity is drastically reduced as the film becomes sub-100 nm, to make it a race between the thinnest possible metal thickness experimentally achievable vs the conductivity reduction. Here, we demonstrate a near-50% absorption at a gold film thickness of 6.5 nm, with conductivity much reduced from the bulk value, down to the range of 10^6^ Siemens per meter. Studying the effect of the substrate thickness, we found that the common cover glass, with its thickness much smaller than the wavelength, achieves symmetric absorption of 44%, implying that a pseudo-free-standing limit is achieved. Our work may find applications in infrared sensing as in bolometers and biomedical sensing using microwaves.

## Introduction

The necessity of optical absorbers has spurred significant research activity which have numerous applications in photocatalytic, Photovoltaic solar cell, optical sensing, and photo electrochemical^1–7^, from the ultraviolet to the radio frequency spectral range^8–10^. Various materials including metastructures and metasurfaces have been discovered, such as 2D materials [e.g., Graphene]^11–14^, phase transition materials [e.g., vanadium dioxide (VO_2_)]^15–17^, semiconductors such as germanium (Ge) on metallic substrate^18,19^, metal- dielectric composites^20^, which can absorb nearly 100% of the incident light. However, synthesis of these materials involves chemical reactions and nano scale patterning with considerably high fabrication costs. Along with this, it needs multi-layer coating to maximize the absorption of electromagnetic wave^21,22^.

On the other hand, homogeneous thin metal film absorbers, while unable to reach maximum absorption of the aforementioned materials, still can enable near-50% absorption of electromagnetic waves without any costly micro or nano patterning, independent of frequency. The maximum absorption of 50% for free standing metal film is reached when the sheet resistance is half the vacuum impedance,

1 $${\mathbf{R}}_{\mathbf{s}}\equiv \frac{1}{{\varvec{\upsigma}}\mathbf{h}}=\frac{{\mathbf{Z}}_{0}}{2}=\frac{1}{2{{\varvec{\upvarepsilon}}}_{0}\mathbf{c}}=\frac{1}{2}\sqrt{\frac{{{\varvec{\upmu}}}_{0}}{{{\varvec{\upvarepsilon}}}_{0}}}=188{\varvec{\Omega}},$$

where s is the metal conductivity, h the film thickness, and ε_0_ and μ_0_ vacuum permittivity and permeability respectively^23–25^. This significant absorption is of orders of magnitudes larger than the thick film limit, where absorption is only of the order of 0.1% at the microwave frequency, given by $\frac{4{\varvec{\uppi}}{\varvec{\updelta}}}{{\varvec{\uplambda}}}$ where d is the skin depth and l the wavelength. Using bulk conductivity of gold, we need film thickness of 1.3 Å to satisfy Eq. (1). However, since metal conductivity reduces drastically as the film gets thinner, we may reach this limit when the thickness becomes sub-10 nm^24,25^. In an actual sample, the film is deposited onto a substrate which inherently affect the absorption.

In this work, we study absorption of electromagnetic waves by a 6.5 nm thick gold film grown on a cover glass. By attaching a number of cover glasses on the substrate side, the substrate-thickness effect is also studied, together with the effect of the side of the incidence. Owing to the large wavelength of the electromagnetic wave used (2 cm) relative to the cover glass thickness (0.013 cm = 130 mm), as-grown sample achieves a free-standing limit, achieving a 44% absorption for either side illumination. In general, substrate-side illumination results in stronger absorption, resulting in over 50% absorption for substrate thickness of a few millimeters. Our experiments are in good agreements with an analytical theory.

## Theoretical framework

### Three-layer system

**Figure 1:**
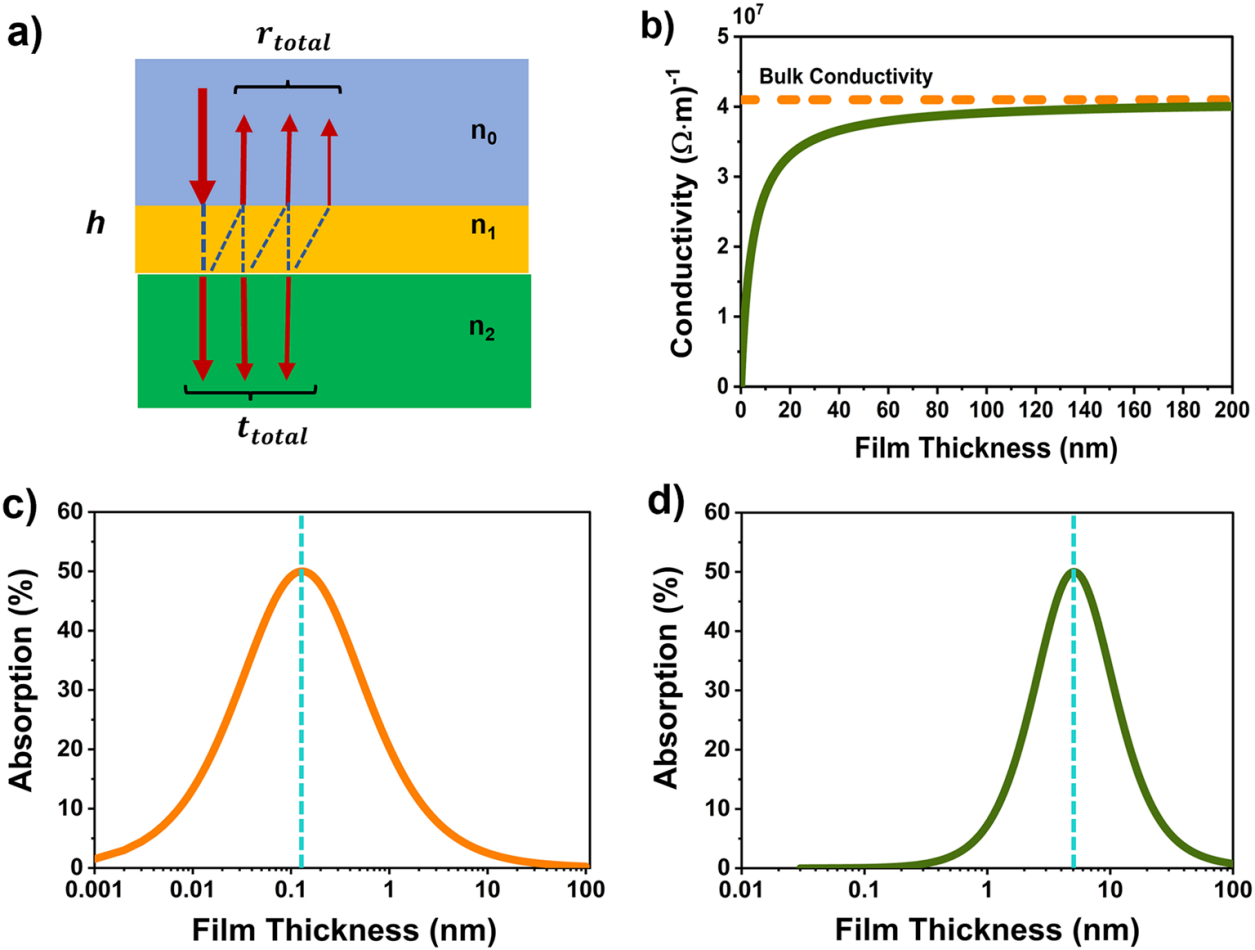
**(a)** Graphic description of the three-layer ultra-thin gold film, where a film of thickness h with complex refractive index ${\mathrm{n}}_{1}={\mathrm{n}}_{\mathrm{m}}$ is embedded between ${\mathrm{n}}_{0}$ and n_1_. **(b)** The dependence of conductivity on gold film thickness (the axis of the conductivity is in the log scale). Conductivities are calculated from supplementary information S1 Eq. (2), using the value of bulk conductivity of the thick gold film $4.1\times {10}^{7}{(\mathrm{\Omega m})}^{-1}$ (dashed yellow line). Absorbance varying thickness of the thin film gold optimized for normal-incidence absorption in free standing limit ${\mathrm{n}}_{0}={\mathrm{n}}_{2}=1$ where, **(c)** $\upsigma$ is the bulk conductivity, and **(d)** the conductivity defined by the (**b**) (the axis of the film thickness is in the log scale and dashed blue line indicate h = h_0_).

At first, we derived an analytical solution of a three-layer system in which a thin film of thickness h and refractive index of n_1_ is sandwiched within top layer and bottom layer having refractive indices n_0_ and n_2_ respectively, shown in Fig. 1a. For simplicity, we assumed the perpendicular incidence of electromagnetic waves on the sample. The total amplitudes are obtained by summing the amplitudes of an infinite number of components from multiple reflection and transmission (although only three are shown in Fig. 1a). With applying appropriate boundary condition at the interface^26,27^, we deduced the analytical expression for the transmission and reflected wave amplitudes which are given by

2 $$\mathrm{t}=\frac{4{\mathrm{n}}_{0}{\mathrm{n}}_{1}{\mathrm{e}}^{\mathrm{i\varphi }}}{\left({\mathrm{n}}_{0}+{\mathrm{n}}_{1}\right)\left({\mathrm{n}}_{1}+{\mathrm{n}}_{2}\right)+({\mathrm{n}}_{0}-{\mathrm{n}}_{1})({\mathrm{n}}_{1}-{\mathrm{n}}_{2}){\mathrm{e}}^{2\mathrm{i\varphi }}},$$

3 $$\mathrm{r}=\frac{\left({\mathrm{n}}_{0}-{\mathrm{n}}_{1}\right)\left({\mathrm{n}}_{1}+{\mathrm{n}}_{2}\right)+({\mathrm{n}}_{0}+{\mathrm{n}}_{1})({\mathrm{n}}_{1}-{\mathrm{n}}_{2}){\mathrm{e}}^{2\mathrm{i\varphi }}}{\left({\mathrm{n}}_{0}+{\mathrm{n}}_{1}\right)\left({\mathrm{n}}_{1}+{\mathrm{n}}_{2}\right)+({\mathrm{n}}_{0}-{\mathrm{n}}_{1})({\mathrm{n}}_{1}-{\mathrm{n}}_{2}){\mathrm{e}}^{2\mathrm{i\varphi }}}$$

where $\mathrm{\varphi }=\frac{\mathrm{\omega h}}{\mathrm{c}}{\mathrm{n}}_{1}$, h is the thickness of the metal film, c is speed of light in vacuum, ω = 2πf, f is the frequency of the electromagnetic wave. The complex refractive index of the metal is given by ${\mathrm{n}}_{1}=\sqrt{(1+\mathrm{i}\frac{\upsigma }{\upomega {\upvarepsilon }_{0}})}$. When the thickness of the film is much smaller than wavelength and also the skin depth: $\frac{\mathrm{h}}{\uplambda }\ll 1; \frac{\mathrm{h}}{\updelta }\ll 1$, the transmission and reflection amplitude are expressed as

4 $$\mathrm{t}=\frac{\frac{{\mathrm{n}}_{0}+{\mathrm{n}}_{2}}{2}}{\frac{{\mathrm{n}}_{0}+{\mathrm{n}}_{2}}{2}+\frac{\mathrm{h}}{{\mathrm{h}}_{0}}},\mathrm{ r}=\frac{\frac{{\mathrm{n}}_{0}-{\mathrm{n}}_{2}}{2}-\frac{\mathrm{h}}{{\mathrm{h}}_{0}}}{\frac{{\mathrm{n}}_{0}+{\mathrm{n}}_{2}}{2}+\frac{\mathrm{h}}{{\mathrm{h}}_{0}}}$$

where ${\mathrm{h}}_{0}=\frac{2{\upvarepsilon }_{0}\mathrm{c}}{\upsigma }$ is characteristic thickness of the metal film which is defined by conductivity. When the thickness of the film is much smaller than the wavelength, the transmission and reflection amplitude are independent of frequency. Finally, the absorption of this system can be calculated by using the following equation

5 $$\mathrm{A}=1-{\mathrm{r}}^{2}-\frac{{\mathrm{n}}_{2}}{{\mathrm{n}}_{0}}{\mathrm{t}}^{2}=\frac{2{\mathrm{n}}_{0}{\mathrm{h}}_{0}\mathrm{h}}{{(\frac{{\mathrm{n}}_{0}+{\mathrm{n}}_{2}}{2}{\mathrm{h}}_{0}+\mathrm{h})}^{2}}$$

From the reflected amplitude and absorption expression, it is apparent that the amplitudes are strongly dependent on the direction of the incidence wave. That means, the absorption from air side incidence could be much different than that from the substrate side incidence as the refractive indices are exchanged. For a freestanding thin film, ${\mathrm{n}}_{0}={\mathrm{n}}_{2}=1$, the absorption can be expressed by

6 $$\mathrm{A}=\frac{2{\mathrm{h}}_{0}\mathrm{h}}{{({\mathrm{h}}_{0}+\mathrm{h})}^{2}}$$

Figure 1c shows absorption peaking at an unachievable thickness of 1.3 $\dot{\mathrm{A}}$. Luckily, when the film gets thinner, the conductivity decreases due to the scattering of electrons at one or both film surfaces and internal grain boundaries^28–30^. Figure 1b presents the conductivity with respect to the thin film thickness using equation (S2) of the Supporting Information (S1). The absorption profile for assuming conductivity as displayed in Fig. 1b, is shown in Fig. 1d.

### Four-layers system

**Figure 2:**
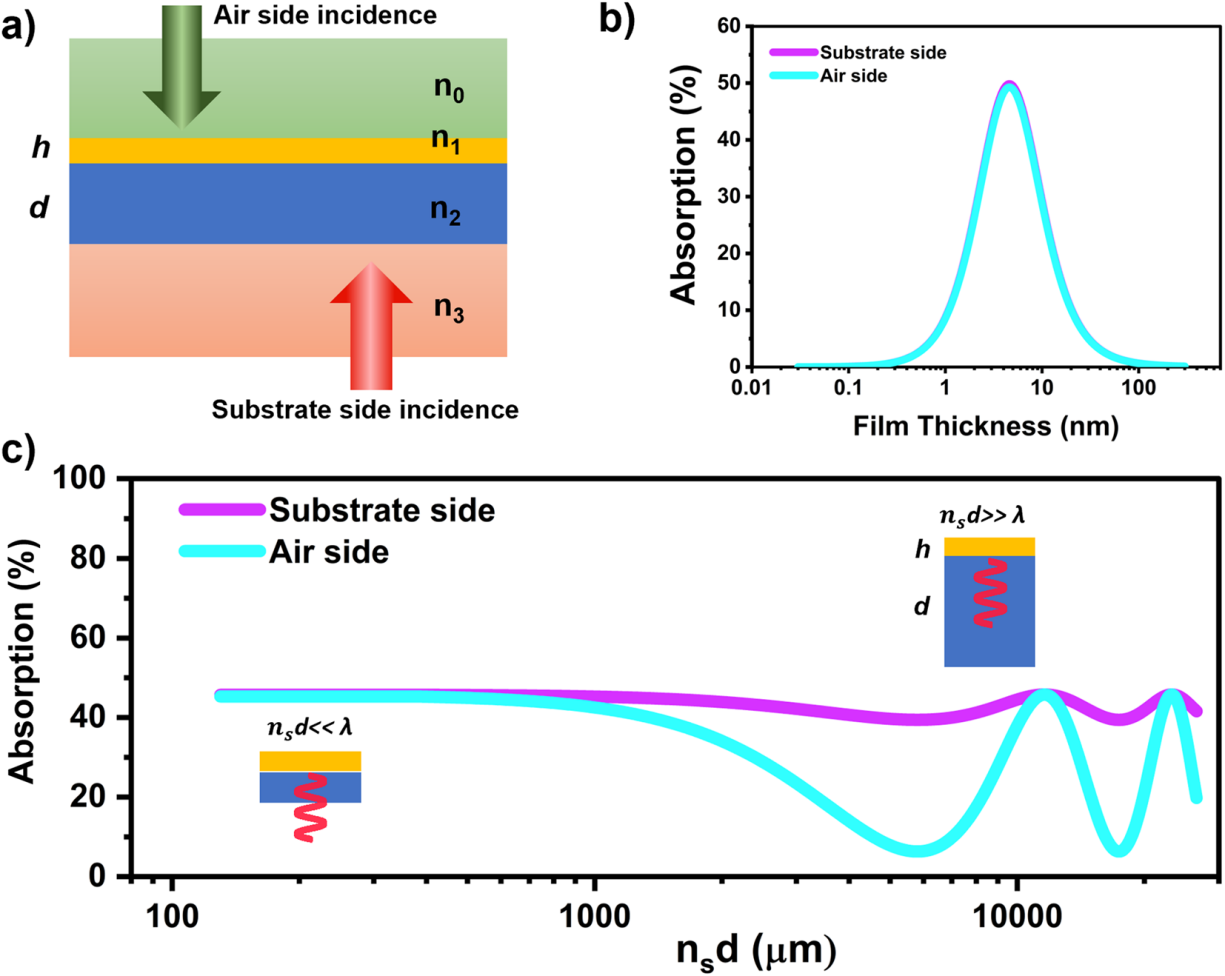
(**a**) Graphic description of the four-layer system for both air side and substrate side incident. (**b**) Absorption spectra by the four-layer system when the thickness of the substrate is very small compared to the wavelength (the axis of the film thickness is in the log scale), (**c**) absorption of the 6.5 nm gold film against the effective thickness of the glass substrate (n_s_d) in log scale (the insets show the schematic of thin and thick substrate related to the microwave wavelength).

Practically, it is hard to achieve a freestanding ultra-thin metal film which would have a maximum absorption; the thin film is often supported with substrate. Here, we put forward a theoretical framework of a four-layer system in which the thin-film on substrate with thickness h and d, respectively is sandwiched with top layer and bottom layer having refractive indices n_o_ and n_3_, as shown in Fig. 2a. By using this four-layered system, we included the effect of substrate analytically. We used transfer matrix method to find out the general expression of transmitted and reflected amplitude via perpendicular incidence of electromagnetic waves through the sample, which are given below^31,32^.

7 $$\mathrm{t}=\frac{{\mathrm{t}}_{01}{\mathrm{t}}_{12}{\mathrm{t}}_{23}{\mathrm{e}}^{\mathrm{i}({\mathrm{\varphi }}_{1}+{\mathrm{\varphi }}_{2})}}{1+{\mathrm{r}}_{01}{\mathrm{r}}_{12}{\mathrm{e}}^{\mathrm{i}2{\mathrm{\varphi }}_{1}}+({\mathrm{r}}_{12}+{\mathrm{r}}_{01}{\mathrm{e}}^{\mathrm{i}2{\mathrm{\varphi }}_{1}}){\mathrm{r}}_{12}{\mathrm{e}}^{\mathrm{i}2{\mathrm{\varphi }}_{2}}}$$

8 $$\mathrm{r}=\frac{{\mathrm{r}}_{01}+{\mathrm{r}}_{12}{\mathrm{e}}^{\mathrm{i}2{\mathrm{\varphi }}_{1}}+({{\mathrm{r}}_{01}\mathrm{r}}_{12}+{\mathrm{e}}^{\mathrm{i}2{\mathrm{\varphi }}_{1}}) {{\mathrm{r}}_{23}\mathrm{e}}^{\mathrm{i}2{\mathrm{\varphi }}_{2}}}{1+{\mathrm{r}}_{01}{\mathrm{r}}_{12}{\mathrm{e}}^{\mathrm{i}2{\mathrm{\varphi }}_{1}}+({\mathrm{r}}_{12}+{\mathrm{r}}_{01}{\mathrm{e}}^{\mathrm{i}2{\mathrm{\varphi }}_{1}}){\mathrm{r}}_{12}{\mathrm{e}}^{\mathrm{i}2{\mathrm{\varphi }}_{2}}}$$

Where ${\mathrm{t}}_{\mathrm{jk}}=\frac{2{\mathrm{n}}_{\mathrm{j}}}{({\mathrm{n}}_{\mathrm{j}}+{\mathrm{n}}_{\mathrm{k}})}$ and ${\mathrm{r}}_{\mathrm{jk}}=\frac{{(\mathrm{n}}_{\mathrm{j}}-{\mathrm{n}}_{\mathrm{k}})}{({\mathrm{n}}_{\mathrm{j}}+{\mathrm{n}}_{\mathrm{k}})}$ are the Fresnel transmission and reflection coefficients, ${\mathrm{\varphi }}_{1}=\frac{\mathrm{\omega h}}{\mathrm{c}}{\mathrm{n}}_{1}$ and ${\mathrm{\varphi }}_{2}=\frac{\mathrm{\omega h}}{\mathrm{c}}{\mathrm{n}}_{2}$. ${\mathrm{n}}_{1}$ and ${\mathrm{n}}_{2}$ are the refractive indices of metal film and substrate, respectively, which for air side incidence ${\mathrm{n}}_{1}={\mathrm{n}}_{\mathrm{m}}$ and ${\mathrm{n}}_{2}={\mathrm{n}}_{\mathrm{s}}$ , and for substrate side incidence ${\mathrm{n}}_{1}={\mathrm{n}}_{\mathrm{s}}$ and ${\mathrm{n}}_{2}={\mathrm{n}}_{\mathrm{m}}$. Figure 2b show the limit of very thin substrate when ${\mathrm{n}}_{0}={\mathrm{n}}_{3}=1$, which is in a good agreement of freestanding limit (Fig. 1d)^32,33^. Figure 2c shows absorption of a thin film gold (thickness 6.5 nm) for varying effective substrate thickness ${\mathrm{n}}_{\mathrm{s}}$ d with incident wavelength fixed at 2 cm. When substrate thickness is very small compared to the wavelength, the absorption reaches its maximum value of < 50%. Moreover, the absorption is independent of whether air or substrate side incidence is chosen. It can be inferred that the sample shows the behavior of a freestanding film when d <  < λ. However, as the substrate thickness is increased, either side absorption decrease, with the airside decreasing much faster due the increasing the effect of substrate with refractive index more that the refractive index of the air. Additional maxima for higher values of ${\mathrm{n}}_{\mathrm{s}}$ d originate from the Fabry Perot modes of electromagnetic waves inside the thick substrate. Similar trends of the free-standing limit (when the substrate thickness is very small w.r.t. wavelength) and the additional absorption maxima can be gleaned also from the reflection and transmission data as shown in the Supporting Information (S2).

### Experimental verification

**Figure 3:**
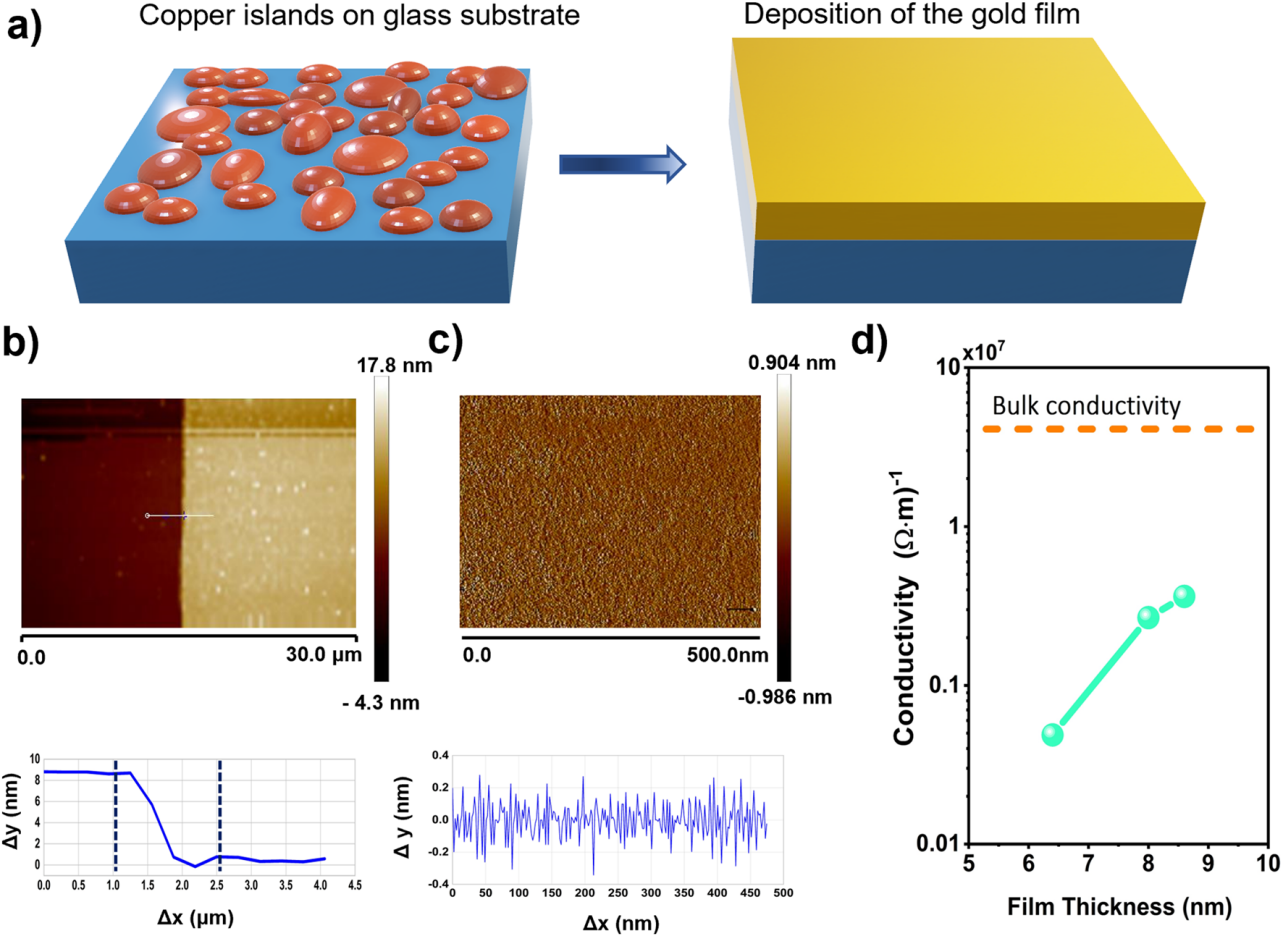
**(a)** Schematic diagram of the fabrication of the uniform ultra-thin film gold on top of the glass substrate. **(b)** AFM image of a step with 7.98 nm thickness, fabricated of mass-equivalent thickness h = 6.5 nm grown on 130 μm glass substrates, with a copper seed layer about 1 nm. **(c)** Surface morphology of the seeded gold with the roughness about 0.2 nm shows continuous and relatively smooth films. **(d)** D.C. conductivity for different thicknesses of film, measured by four probe technique (the axis of the conductivity is in the log scale and dashed yellow line shows bulk conductivity of thick gold film ($4.1\times {10}^{7}{(\mathrm{\Omega m})}^{-1}$).

To check the validity of theoretical framework in achieving a maximum absorption of 50% with a thin metal film supported by substrate, we fabricated ultra-thin gold metal on cover glass substrate of thickness 130 μm. The fabrication procedure to make uniform thin film by using copper seeding technique is reported elsewhere^34–36^ and explained in detail in the methods section. The schematic diagram of the fabrication is shown in Fig. 3a. The key idea is deposition of the 1 nm copper seed layer on glass substrate before the gold deposition. The exposed copper seeds layer to air are likely to undergo oxidation to have negligible effect of optical and electrical conductivity. We fabricated different thickness of thin film ranges from 6.5 to 10 nm on cover glass; films below 6.5 nm thickness failed to form proper films.

**Figure 4:**
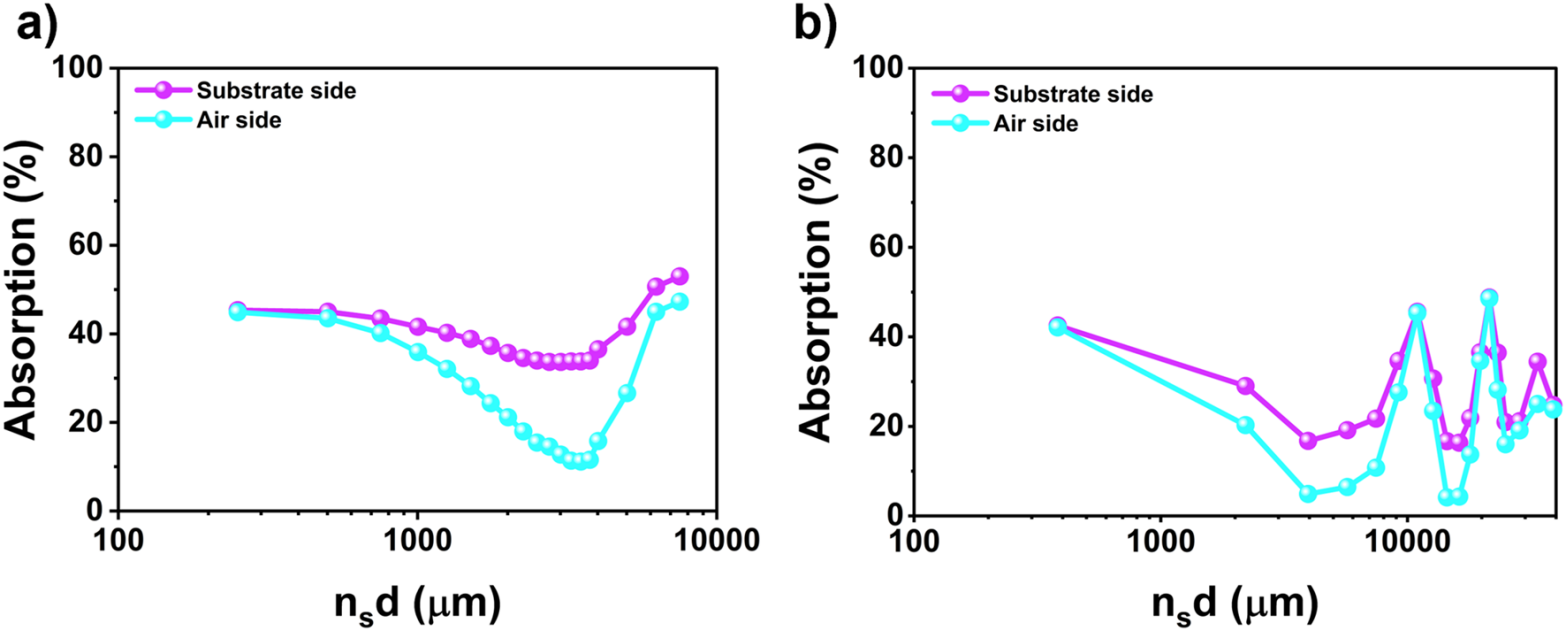
Absorption amplitude of 6.5 nm gold film on against effective thickness n_s_d of the **(a)** glass and **(b)** silicon substrate. Note that the absorptions are measured from both air side and sample side incident. The extinction peak for absorption is in the limit of thick substrate which is discussed before in the main text. Absorption oscillates when the substrate thickness increase, due to the Fabry–Perot interference effects (all the axis of the substrate thickness are in the log scale).

The thinnest film, 6.5 nm thick achieved the highest absorption, whose atomic force microscopy (AFM) measurement is shown in Fig. 3b,c. An average geometrical thickness of the film layer 7.98 nm is measured with the roughness of about 0.2 nm. Subtracting the thickness of the copper oxide used as a seed layer of about 1.4 nm, we estimated the thickness of the gold film to be around 6.5 nm^35–37^. We checked the D.C. conductivity of various thickness film by Keithley Source meter 2450. Figure 3d represents the respective data. Although samples are very thin, good conductivities of the order of 10^6^ S per meter (${(\mathrm{\Omega m})}^{-1}$) are obtained. For the thinnest sample i.e., 6.5 nm thin film, the conductivity is found to be $0.09\times {10}^{7}{(\mathrm{\Omega m})}^{-1}$.The respective experimental characteristic thickness (${\mathrm{h}}_{0}=\frac{2{\upvarepsilon }_{0}\mathrm{c}}{\upsigma }$) is calculated to be 5.9 nm which is close to the actual film thickness. With this thickness of film deposited onto a very thin substrate (in this case cover glass of thickness 130 μm), we expect that the effect of the substrate will be negligible when the incident wavelength is very long, and a near-maximum absorption can be achieved. That’s exactly what we observed when we measured the absorption of the sample in microwave frequency of 12–18 GHz (see Supporting Information (S3) for the details of the microwave spectroscopy)^37,38^. Since the absorption is frequency independent, we focus on a single frequency of 15 GHz with wavelength of 2 cm (Fig. 4a). Absorption from the substrate side and the air side incidents reached 44.9% and 45.3%, respectively for the as-grown sample. Nearly equal absorptions for both sides prove that the sample is working in the free-standing limit (${\mathrm{n}}_{\mathrm{s}}$ d <  < λ). Absorption is still less than 50% of the free-standing limit maximum, because the thickness of our ultra-thin film gold could not reach the characteristic critical thickness of ${\mathrm{h}}_{0}$. When we increase the thickness of substrate simply by piling up a number of cover glasses on the substrate side, the absorption at 15 GHz starts to decrease followed by addition maxima due to Fabry Perot effect as described in the theoretical framework (Fig. 2c). (Fig. S4a,b in supplementary information show the reflection and transmission amplitude, respectively, for different thickness of the glass substrate). As a control experiment, we fabricate ultra-thin metallic films on silicon substrate and increase the effective thickness ${\mathrm{n}}_{\mathrm{s}}$ d. Absorption from the substrate side and the air side incidents for thin film on silicon substrate are shown in Fig. 4b. Due to the interference effects, reflection, transmission, and absorption oscillate when the substrate thickness increase (see supplementary Information S4c,d for reflection and transmission amplitude, respectively). Increasing the thickness of the silicon substrate, some scattering happened due to the air layers compressed between the added substrates. However, the absorption of the ultra-thin film is in the good agreement with the cover glass substrate as we expected. Our advantages are (1) we have no patterns; (2) thereby, our spectral response is very broad as explained by a comparison provided in Table S1 in supplementary information.

## Conclusions

In summary, we described theoretical framework on achieving a maximum absorption for a thin film supported with a substrate. We reported that the characteristic thickness ${\mathrm{h}}_{0}=\frac{2{\upvarepsilon }_{0}\mathrm{c}}{\upsigma }$ is a vital physical parameter of the film which can be tuned to achieve the maximum value of absorption. When substrate thickness is very small compared to the incident wavelength, the sample behaves as a freestanding. Experimentally, we fabricated 6.5 nm thickness of ultra-thin film on 130 μm thick substrate. For microwave frequency, it shows a near maximum absorption of 44%. Our work may be useful various applications in photonics, optoelectronics etc.

## Methods

As ultra-thin metal layer tends to form islands rather than homogenous film, obtaining homogeneous thin films with sub nanometer thickness can be of profound importance. The new deposition method which called copper seeding technique^34–36^ allows us to reach minimum thickness of the gold and avoids the problem of island-like growth of unseeded gold at very small thickness. In this technique 1 nm copper is deposited using e beam evaporator system (KVE-E400) as a seed layer, which remain in the air, are likely to undergo oxidation to have negligible effect of optical and electrical conductivity. On top of the thin copper oxide layer, different thicknesses of the gold layer are deposited on a Glass substrate. 6.5 nm, 8 nm, and 8.5 nm gold are deposited at a rate of 0.3 $\dot{\mathrm{A}}$/s under a pressure of 10^6^ torr without rotation of the substrate.

## Supplementary Information

Supplementary Information.

## Data Availability

The data that support the findings of this study are available from the corresponding author upon reasonable request.
