# Supplementary material for: Near-maximum microwave absorption in a thin metal film at the pseudo-free-standing limit
Source: Sci Rep. 2022 Nov 1;12:18386. doi: 10.1038/s41598-022-23119-7 (PMC9626603; doi:10.1038/s41598-022-23119-7)
Supplement: Supplementary file 1 — Supplementary Information. [file 41598_2022_23119_MOESM1_ESM.pdf]

Supplementary information:

Near-Maximum Microwave Absorption in a Thin Metal Film at the Pseudo-Free-Standing Limit

Mahsa Haddadi M.[1,#], Bamadev Das[1,#], Jeeyoon Jeong[2],Sunghwan Kim[1], Dai-Sik Kim[1,*]

[1] Department of Physics and Quantum Photonics Institute, Ulsan National Institute of Science and Technology (UNIST), Ulsan 44919, Republic of Korea,
[2] Department of Physics and Institute of Quantum Convergence Technology, Kangwon National University, 1 Gangwondaehak-gil, Chuncheon-si, Gangwon-do 24341, Republic of Korea
*Correspondence: (D.S.K) daisikkim@unist.ac.kr
[#] M.H.M. and B.D. contributed equally.

S1) Conductivity vs thickness of ultra-thin metal film

An exponential decrease in the metal conductivity is observed for the thinner films. Fuchs (1938) and Sondheimer (1952) made use of Boltzmann distribution equation which described that the electron energy changes due to applied fields and collisions [1-3]

$$\frac{\sigma}{\sigma_0} = 1 - \frac{3l}{2h}(1-p)\int_1^{\infty}\left(\frac{1}{t^3}-\frac{1}{t^5}\right)\left(\frac{1-e^{-\frac{ht}{l}}}{1-pe^{-\frac{ht}{l}}}\right)dt, \quad \text{(S1)}$$

Where t is the integration variable, h is metal film thickness, σ_0 and l are bulk conductivity and mean free path of the electrons inside the bulk metals, respectively. p the probability that an electron will be reflected specularly upon scattering from one of the surfaces. Typical values for p are 0 for polycrystalline films and 0.5 for single crystal films. For very small h the equation become

$$\frac{\sigma}{\sigma_0} = \left[1 + \frac{0.375(1-p)sl}{h} + \frac{1.5Rl}{(1-R)g}\right]^{-1} . \quad \text{(S2)}$$

where S is a roughness parameter that equals 1 in perfect metal with flat interfaces and more than 1 when the roughness of the surface increase. R is the scattering coefficient, illustrating the scattering of electrons at the grain boundaries. g is the average grain size. Characteristics such as roughness can be included in the parameter p, since they directly related to the way electrons are scattered.

S2) Theoretical transmission and reflection amplitude of the thin film gold in four-layer system

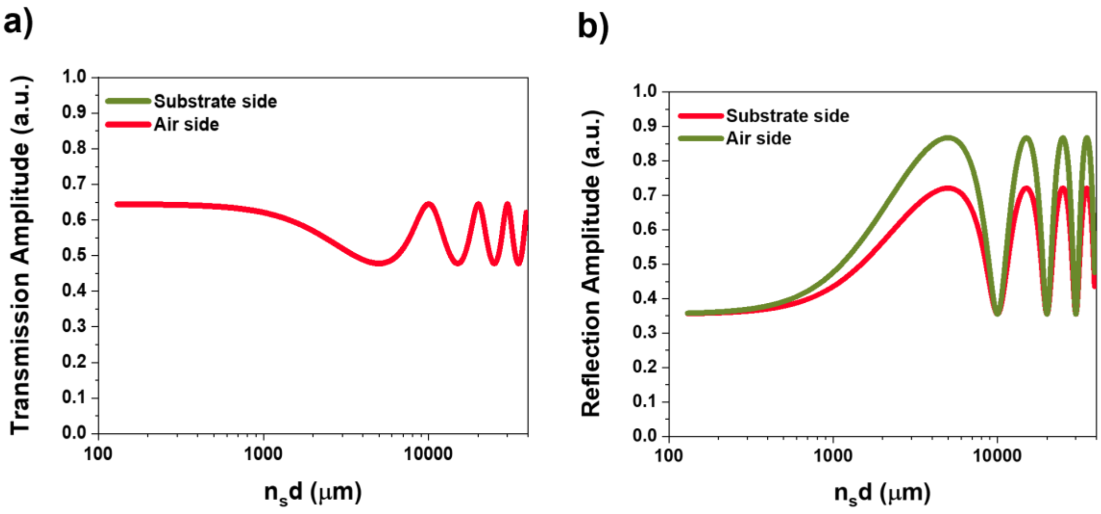

Figure S2. (a) Transmitted amplitude, and (c) reflected amplitude of 6.5 nm gold film against effective thickness of the substrate ($n_s d$). Note that the transmission and reflection are calculated from both air side and sample side incident (All the axis of the substrate thickness are in the log scale). Transmission from the substrate side incidence overlapped with the air side incidence.

S3) Microwave setup

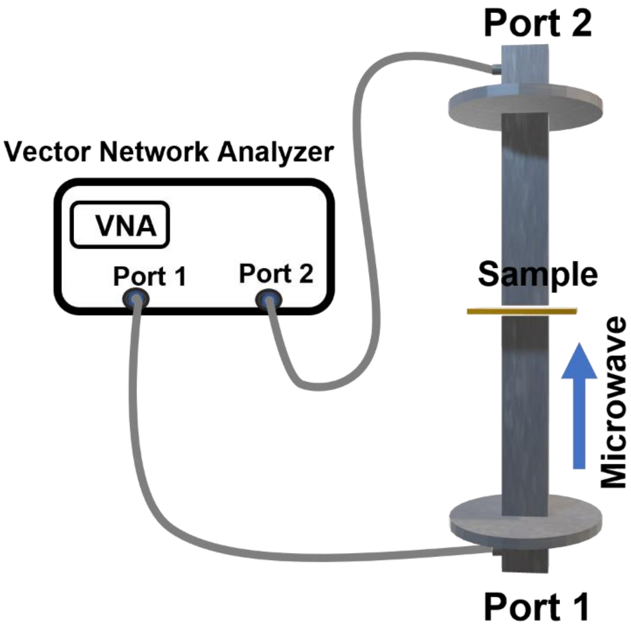

Figure S3. Details of the microwave spectroscopy setup. Schematic diagram of the microwave transmission and reflection setup. The above figure shows our waveguide setup for Ku band (12-18 GHz) vertically aligned with the sample in the middle. A pair of open-ended rectangular waveguide (62EWGN) connected with network analyzer (E5063A) was used for the microwave measurement. The aperture size for Ku band is 15.80 mm by 7.90 mm, which supports the TE10 mode. For calibration, a thru reflect-line calibration technique was performed [4,5].

S4) Experimental reflection amplitude and transmission amplitude of the thin film gold on varying substrate thickness

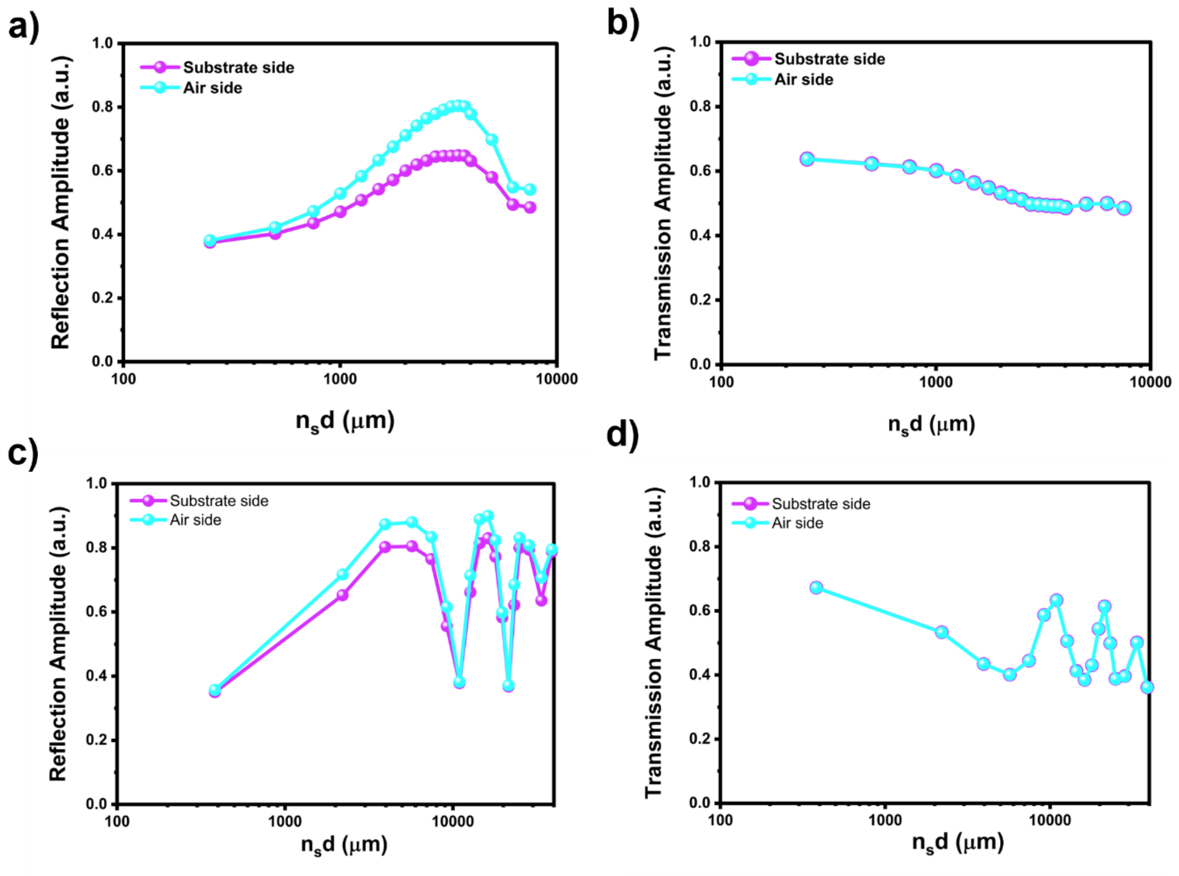

Figure S4. (a) Reflected amplitude and **(b)** transmitted amplitude of *6.5 nm* gold film against effective thickness $n_s d$ of the glass substrate. **(c)** Reflected amplitude and **(d)** transmitted amplitude of *6.5 nm* gold film against effective thickness $n_s d$ of the silicon substrate. Note that the transmission and reflection are measured from both air side and sample side incident. Reflection, and transmission oscillate when the substrate thickness increase, due to the Fabry-Perot interference effects. (All the axis of the substrate thickness are in the log scale). Transmission from the substrate side incidence overlapped with the air side incidence.

Table (S1)

System	Materials	Maximum absorption (%)	Wavelength	Limitations	Referenc e
Ultra-thin film	Gold	45.3	Microwave	Maximum absorption 50%	This work
Multilayer based on 2D materials	Graphene	77.6	Visible ~ mid-infrared	Costly nanofabrication, Low throughput	[6,7]
Multilayer based on phase transition materials	VO_2	90 ~ 30	Mid-infrared	Multilayer deposition, Costly nanofabrication, and Thick	[8,9]
Multilayer with Metal reflector and lossless dielectric layer	MDS	99.8	Visible	Multilayer deposition, Costly nanofabrication, Low absorption in longer wavelength, and Thick	[10]
Multilayer with nano particles	M-D-MNP	81	Visible	Multilayer deposition, Costly nanofabrication, and Thick	[11]
Multilayer with Metal reflector	M-S-M	95.5	Visible	Multilayer deposition, Costly nanofabrication, Low absorption in longer wavelength, and Thick	[12]

M: metal
S: Semiconductor
D: Dielectric
MNP: metal-nanoparticles

Table S1. Comparative study of different methods and materials with respect to their limitations.

References

1. Campbell, D. S. & Morley, A. R. Electrical conduction in thin metallic, dielectric and metallic-dielectric films. Reports on Progress in Physics 34, 283-368 (1971).
2. Ding, G., Clavero, C., Schweigert, D. & Le, M. Thickness and microstructure effects in the optical and electrical properties of silver thin films. AIP Advances 5, 117234 (2015).
3. Kasap, S., P.C., Springer Handbook of Electronic and Photonic Materials. Springer Handbooks. (2007).
4. Das, B., Yun, H. S., Park, N., Jeong, J. & Kim, D.-S. A Transformative Metasurface Based on Zerogap Embedded Template. Advanced Optical Materials 9, 2002164 (2021).
5. Kim, D. et al. Topology-Changing Broadband Metamaterials Enabled by Closable Nanotrenches. Nano Letters 21, 4202-4208 (2021).
6. Li, Q., Lu, J., Gupta, P. & Qiu, M. Engineering Optical Absorption in Graphene and Other 2D Materials: Advances and Applications. *Advanced Optical Materials* **7**, 1900595 (2019).
7. Pham, P. H. Q. *et al.* Broadband impedance match to two-dimensional materials in the terahertz domain. *Nature Communications* **8**, 2233 (2017).
8. Kocer, H. *et al.* Intensity tunable infrared broadband absorbers based on VO2 phase transition using planar layered thin films. *Scientific Reports* **5**, 13384 (2015).
9. Rajeswaran, B., Pradhan, J. K., Ramakrishna, S. A. & Umarji, A. M. Thermochromic VO2 thin films on ITO-coated glass substrates for broadband high absorption at infra-red frequencies. *Journal of Applied Physics* **122**, 163107 (2017).
10. Song, H. *et al.* Nanocavity Enhancement for Ultra-Thin Film Optical Absorber. *Advanced Materials* **26**, 2737-2743 (2014).
11. Zhang, N. *et al.* Refractive index engineering of metal-dielectric nanocomposite thin films for optical super absorber. *Applied Physics Letters* **104**, 203112 (2014).
12. Lee, K.-T., Ji, C. & Guo, L. J. Wide-angle, polarization-independent ultrathin broadband visible absorbers. *Applied Physics Letters* **108**, 031107 (2016).
